# Does repeated autoclave sterilization cause changes in the color and fragility of fiberglass reinforced resin crowns?

**DOI:** 10.1186/s12903-023-03254-x

**Published:** 2023-08-01

**Authors:** Ebru Delikan, Seçil Çaliskan, Ahmet Çalışkan, Canan Özdemir

**Affiliations:** 1 Department of Pediatric Dentistry, Faculty of Dentistry, Nuh Naci Yazgan University, Kayseri, Turkey; 2 Department of Pediatric Dentistry, Faculty of Dentistry, Eskişehir Osmangazi University, Eskişehir, Turkey; 3 Special Uzman Diş Grup Oral and Dental Health Polyclinic, İzmit, Turkey; 4 Private Clinic, Eskişehir, Turkey

**Keywords:** Autoclave sterilization, Color stability, Failure analysis, Fiberglass reinforced resin crowns, Fracture resistance

## Abstract

**Background:**

Since the effects of sterilization on the Fiberglass Reinforced Resin Crowns (FRRCs) is not well-known the aim of current study was to evaluate the effects of autoclave sterilization on the fracture resistance, color stability, and surface composition of FRRCs.

**Methods:**

A total of 48 crowns were used. The crowns were divided into three groups according to the sterilization number: no sterilization (Control Group), one sterilization (Group 1), and four sterilizations (Group 2). The microstructure of the three crowns from each group was evaluated using scanning electron microscopy (SEM) and energy-dispersive X-ray (EDX) spectroscopy. Thirteen FRRCs from each group were first used for color stability testing and then for the fracture resistance analysis. One-way analysis of variance (ANOVA), one-way repeated measures ANOVA, and paired t-tests were used in the statistical analysis.

**Results:**

EDX results revealed that the weight% of surface silicon atoms in group 2 was significantly higher. Some crack lines could be observed on the SEM images. Statistically significant differences were found in color stability following the first and fourth sterilization cycles (p < .01). The increase in the sterilization cycle did not statistically decrease the fracture resistance of the FRRCs (p = .055); however, overall, a decreasing trend was observed in fracture resistance as the sterilization cycle increased.

**Conclusions:**

Autoclave sterilization caused some changes in the surface elemental composition and surface morphology of FRRCs. Avoiding unnecessary FRRC trials is important to reduce the number of sterilizations.

## Background

One of the challenges in pediatric dentistry is restoring of severely damaged primary teeth due to caries. Although restorative materials, such as amalgams, composite resins, and glass ionomers, are used in restorative treatment, the American Academy of Pediatric Dentistry recommends the use of a full-coverage restoration for children with excessive crown destruction [1]. When full coverage is required, stainless steel crowns (SSCs) are the gold standard of treatment because they are extremely durable and inexpensive and require minimum technique sensitivity [2]. However, they present a poor aesthetic appearance with their metallic color, which may be a deterrent for some parents [3]. Open-face SSCs are manufactured as a cosmetic solution to conventional SSCs, but they are time-consuming, and their metal margin compromises aesthetics [4]. Similarly, resin strip crowns provide a superior aesthetic, but have a sensitive technique, are not resistant to chewing forces, and show frequent fractures [5]. Unlike other full-coverage restorations, preformed zirconia crowns with sufficient mechanical strength and durability, good chemical and dimensional stability, and good aesthetics have become very popular in pediatric dentistry [6, 7]. Nevertheless, despite these advantages, they also have some disadvantages, such as being very expensive, requiring excessive preparation, time-consuming adjustment, and causing wear on the opposite tooth [8]. Additionally, salivary contamination during the try-in of the restoration can weaken the bond to the resin cement [9]. Therefore, autoclavable trial crowns are needed, but they entail additional costs.

Fiberglass-reinforced resin crowns (FRRCs) for primary teeth were introduced in 2018 to eliminate these disadvantages. They are prefabricated fiberglass or quartz fibers embedded in composite resin. Fiber provides support and strength to the composite matrix [10]. FRRCs have the advantages of having pleasing aesthetics and durability, and they do not contain metallic restorative materials [11]. These biocompatible crowns replicate the anatomy of natural teeth, and they are bisphenol A-free and autoclavable [12]. FRRCs are available in various sizes. The tooth is prepared to fit the inner surface of the crown. Although crowns are produced under aseptic conditions, trying multiple crowns may be necessary to find an accurate fit since there is no trial crown within the set. Consequently, several crowns can be contaminated during this period. These disused crowns may have to be sterilized multiple times for use in different patients.

There are different sterilization methods to eliminate all forms of microorganisms, including autoclave sterilization, ethylene oxide gas (EOG) sterilization, chemical vapor sterilization, dry-heat sterilization, gamma sterilization, and chemical sterilant. Among these methods, autoclave and EOG sterilization are the most frequently used in dentistry [13]. A typical autoclave sterilization process includes heating, exposure, and cooling phases. These phases causes temperature changes in the samples [14]. And the temperature changes may lead to chemical alterations in material chemistry and morphology. According to a study of conventional and bulk-fill composites, autoclave sterilization improved wettability and increased the amount of filler particles exposed, but it had no discernible impact on surface roughness [14]. However, the effects of autoclave sterilization on FRRCs have not been investigated to date.

The present study experimentally investigated the effects of autoclave sterilization on the surface composition, color stability, and fracture resistance of FRRCs. The null hypothesis is that sterilization cycles do not affect the chemical composition, color stability, or fracture resistance of FRRCs.

## Methods

A total of 48 primary mandibular right first molar FRRCs (Figaro Crowns, Inc., Woodbury, MN, USA) were randomly divided into three groups with 16 crowns in each group. One group served as control (Control Group: no sterilization) and two were experimental (Group 1: one sterilization, and Group 2: four sterilizations; with an interval of a day). The crowns in Group 1 and Group 2 were placed in self-sealing sterile pouches and were sterilized in an autoclave (Eryiğit Steam Sterilizer, Ankara, Turkey) for 20 min at 121℃ under 1-atmosphere pressure. Sterilization was performed by the calibrated researcher and stages were checked with a previous pilot study.

**Fig. 1:**
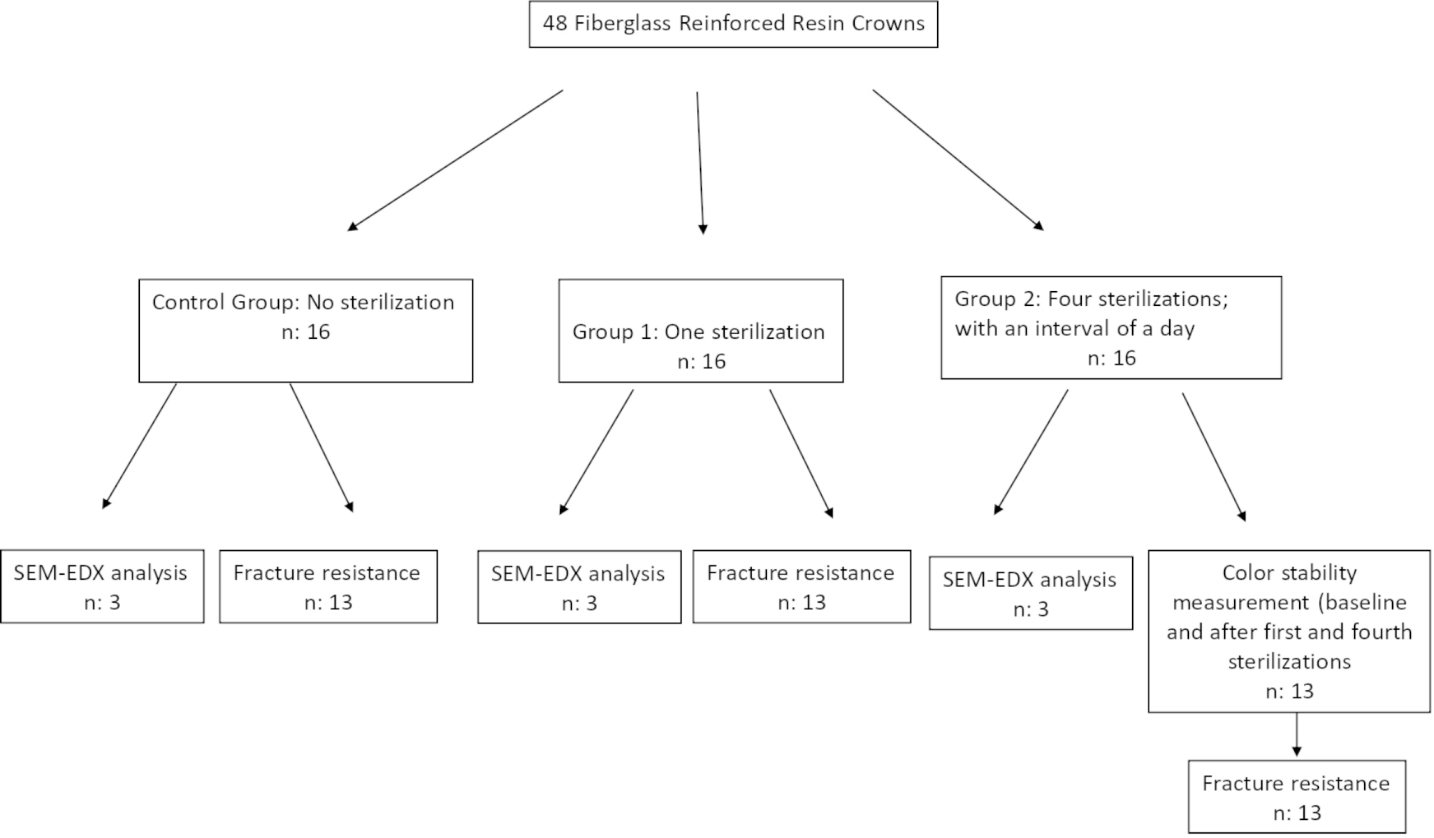
The flow chart of the study

The sample size was determined using power calculation (Version 3.1.9.4, Heinrich Heine, University of Düsseldorf, Düsseldorf, Germany), which gave an estimated power of > 90% with 13 specimens per group. In the estimation, a supposed significance level of 0.05 and an effect size of 0.25 were applied [15]. Since 13 crowns for color stability evaluation and 3 crowns for scanning electron microscopy (SEM) and energy-dispersive X-ray spectroscopy (EDX) analyses would be evaluated, the number of samples in the groups was planned as 16 crowns (Fig. 1).

### Microstructure observation using scanning electron microscopy (SEM) and energy-dispersive X-ray spectroscopy (EDX) analysis

Three crowns from each group (total of nine crowns) were used for SEM (Gemini 500; Zeiss, Oberkochen, Germany) and EDX (EDAX Octane Elect, Amatek, USA) using an acceleration voltage of 20 kV (Fig. 1). Before SEM analysis, the surfaces of the three crowns from each group were mounted on metal stubs and sputter coated with Au/Pd (80% Au, 20% Pd). Surface topographies were observed using SEM at 500×, 1,000×, and 1,500× magnification, following sterilization. The chemical composition of the crowns was determined using EDX.

### Color stability measurement

Color stability measurement was tested on the thirteen crowns in group 2 (Fig. 1). Color change measurement was preferred in this group because measurements could be repeated on the same crowns at the baseline and after 1st and 4th sterilization. Vita EasyShade spectrophotometer (Vita Zahnfabrik GmbH, Bad Säckingen, Germany), calibrated according to the manufacturer’s recommendation before each measurement, was used to analyze the color changes. A single operator performed the baseline color measurements of the crowns (T0), and the measurements were repeated after the first (T1) and fourth sterilizations (T2). A neutral gray background was used for measurement. Each crown was gently dried with tissue paper, and the instrument probe was placed perpendicular to the crown surfaces. Values were recorded based on the Commission Internationale de l’Eclairage L*a*b* system. In this uniform color space, L* indicates lightness, with positive values being whiter and negative being darker; a* indicates positive values being red and negative values being green; and b* indicates positive values being yellow and negative values being blue. Three measurements were performed for each crown surface, and the mean values were calculated. The color change value (ΔE) for each crown was calculated using the formula ΔE (L*a*b*) = ([ΔL*] 2 + [Δa*] 2 + [Δb*]2) ½. To assess color changes clinically acceptability threshold was assumed ΔE = 3.3 based on a previous study [16].

### Fracture resistance measurement

Thirteen crowns of each group were used to evaluate the effect of autoclave sterilization on the fracture resistance of FRRCs (Fig. 1).

For the fracture resistance calculation, a die model (negative replica of a crown) was fabricated using cold-curing acrylic (Orthocryl® Dentaurum, Ispingen, Germany). C-type silicone (Silect Set, Muller-Omicron GmbH & Co., KG, Germany) was used to make a mold, and 39 ortho-resin die models were fabricated and left to be set for 24 h. The obtained die models were tested, and visible undercuts were removed to ensure a passive fit to the crowns. Fiberglass-reinforced resin crowns were cemented on die models using glass-ionomer cement (Ketac, 3 M ESPE, Seefeld, Germany) according to the manufacturer’s instructions. The die–crown units were allowed to set for 24 h. Each dies with a cemented crown was placed in a holder on an Instron test device (MOD Dental, Esetron, Turkey). Two layers of foil sheet were placed in between to achieve homogenous stress distribution and minimization of the transmission of local force peaks. The compressive force was applied along the long axis of the crowns’ mid-occlusal central fossa using a stainless-steel round tip load applicator at a crosshead speed of 1 mm/min until the crown fractured. The load at failure, confirmed by a sharp drop at the load–deflection curve, was recorded using computer software. The maximal fracture loads were recorded in Newtons (N).

### Statistical analysis

Statistical software (SPSS 22; IBM, Armonk, NY, USA) was used for data analysis. The distribution of the data was controlled using the Shapiro-Wilk test. Data were presented as the mean and standard deviation (SD) for the values. The results of the surface chemical composition (C: Calcium, O: Oxygen, Al: Aluminum, Si: Silicon, Cl: Chlorine, Ba: Barium) and fracture resistance were statistically analyzed using a one-way analysis of variance (ANOVA) test. The significant differences among the groups were identified using the Tukey multiple comparison test. Repeated measures ANOVA with Bonferroni post hoc test and a paired t-test were used to compare the color measurements (L* a* b* and ΔE) of the crowns after the sterilization cycles. The level of significance was set to α = 0.05.

## Results

### Surface changes/SEM-EDX analysis

**Fig. 2:**
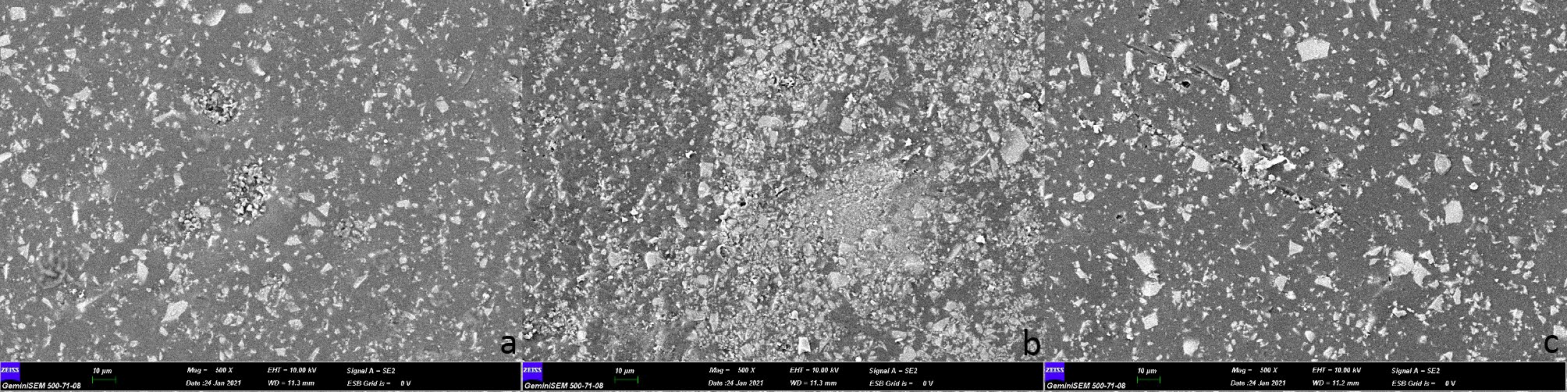
SEM images (500x) of crown surfaces in each group (a: control group, b: group 1, c: group 2). Superficial morphologies were similar in all groups however some crack/ slit lines could be seen in group 3

**Fig. 3:**
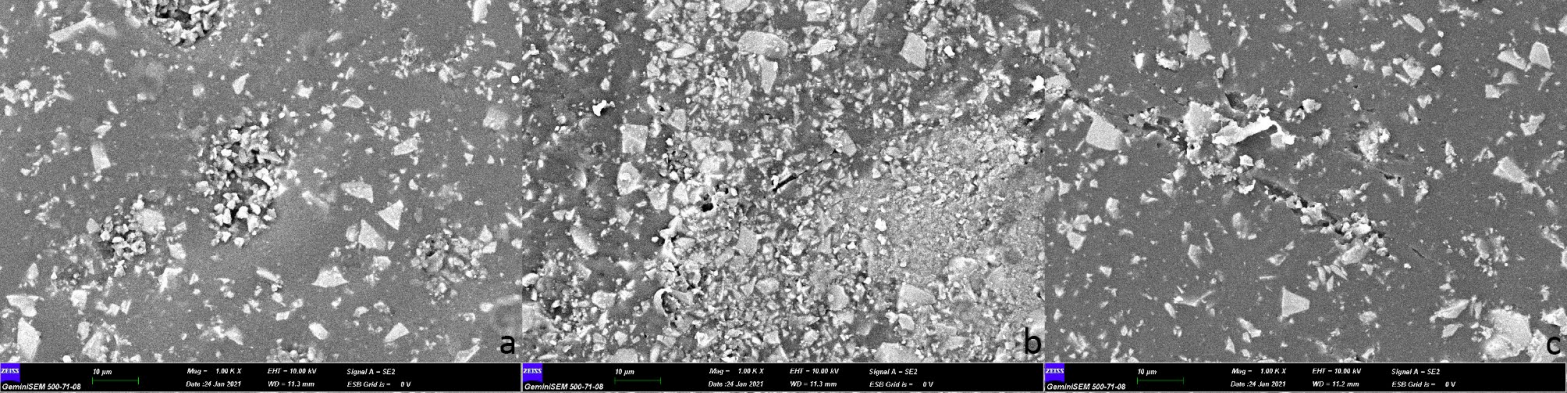
SEM images (1000x) of crown surfaces in each group (a: control group, b: group 1, c: group 2). Crack/ slit lines could be seen in group 1 and more in group 2

**Fig. 4:**
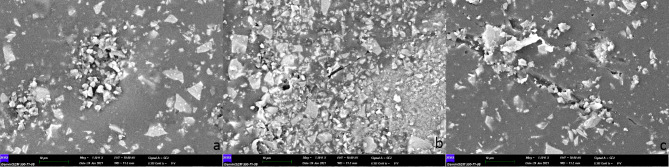
SEM images (1500 x) of crown surfaces in each group (a: control group, b: group 1, c: group 2). No fracture was observed on the crown surfaces in all groups. However, some crack/ slit lines could be clearly seen in group 1 and more in group 2

Three crowns from each group were analyzed using SEM at 500× (Figs. 2), 1,000× (Fig. 3), and 1,500× (Fig. 4) magnification, and no fracture was observed on the crown surfaces.

However, some crack lines were noticed after sterilization. The surface elemental compositions (wt%) of the crowns are shown in Table 1. The presence of silicon atoms on the surface of group 2 was significantly greater than that of group 1 (p = .041). No statistically significant differences were found in the weight% of other surface elements (Table 1).

**Table 1:** Surface elemental composition (wt %) of FRRCs (Mean and standard deviation)

Elemental Composition	Sterilization			*p value* ^*^
	Control Group	Group 1	Group 2	
C	60.14 ± 3.056	49,99 ± 9,49	60.59 ± 1.19	0.113
O	25.01 ± 3.15	27.11 ± 1.98	22.78 ± 2.37	0.195
Al	1.55 ± 0.11	2.14 ± 0.74	1.56 ± 0.15	0.254
Si	5.90 ^a^ ± 0.26	10.37 ^b^ ± 3.00	6.40 ^a,b^ ± 0.64	**0.041**
Cl	1.48 ± 0.17	1.24 ± 0.50	1.55 ± 0.19	0.514
Ba	5.92 ± 0.45	9.15 ± 5.10	7.12 ± 1.01	0.462

* p values are based on the one-way Anova test. Bold numbers represent the statistically signifi-cant difference (p < .05). C: Calcium, O: Oxygen, Al: Aluminum, Si: Silicon, Cl: Chlorine, Ba: Barium. The different lowercase letters represent the statistical difference between the groups on the same line. Control Group: no sterilization, Group 1: one sterilization, Group 2: four sterilizations with an interval of a day

### Color stability

The mean and standard deviation of the L* a* b* and ΔE values of the crowns after the sterilization cycles are presented in Table 2. Fiber-reinforced resin crowns became insignificantly lighter and redder (p > .05) and then became significantly more yellow following the fourth sterilization cycle (p < .01) (Table 2). All crowns exhibited discoloration after sterilization. After the first and fourth sterilizations, a statistically significant difference was found in the ΔE values of the crowns (p < .01). Color changes following the first and fourth sterilizations were above the clinically acceptable value. The highest ΔE values were observed following the fourth sterilization. The color change of FRRCs increased with sterilization.

**Table 2:** L*a*b* and ΔE values of FRRCs

Color measurement periods		Mean ± SD	p-value^*^
	T_0_	78.69 ± 20.47	0.785
L^*^	T_1_	76.82 ± 18.34	
	T_2_	81.43 ± 1.56	
	T_0_	-2.19 ± 1.37	0.165
a^*^	T_1_	-1.97 ± 0.97	
	T_2_	-1.49 ± 1.01	
	T_0_	11.92 ± 2.20	
b^*^	T_1_	17.63 ± 3.63	**< 0.01**
	T_2_	18.99 ± 3.92	
	T_0_/T_1_	5.9580 ± 2.21	**< 0.01**
ΔE value	T_0_/T_2_	7,4851 ± 2,81	

* p values are based on the repeated measures Anova test with Bonferroni post-hoc test. Bold numbers represent the statistically significant difference (p < .05). T0, T1 and T2 indicate the base-line color measurement, the color measurement after the first sterilization and the color meas-urement after the fourth sterilization of the crowns, respectively

### Fracture resistance

The mean fracture resistance values and standard deviations of the crowns are shown in Table 3. No significant difference was found between the fracture resistance values of the groups (p = .055). Although not statistically significant, fracture resistance showed a downtrend decrease with increasing sterilization numbers.

**Table 3:** Fracture resistance (Mean ± standard deviation) of FRRCs

	Groups			*p value* ^*^
	**Control Group**	**Group 1**	**Group 2**	
**Fracture** **Resistance**	1534.54 ± 285.56	1444.85 ± 156.44	1189.23 ± 196.23	0.055

* p values are based on the one-way Anova test and level of significance was set at p < .05. Fracture resistance values were measured in Newtons (N). Control Group: no sterilization, Group 1: one sterilization, Group 2: four sterilizations with an interval of a day

## Discussion

This study evaluated the effect of sterilization cycles on the chemical composition, color stability, and fracture resistance of FRRCs. According to the results obtained, the null hypothesis of the study was rejected in terms of chemical composition and color stability and accepted in terms of fracture resistance.

In vitro study models are frequently used by many researchers to evaluate the mechanical properties of dental materials. It can simulate the oral environment from different perspectives and create a controlled environment that allows the testing of material properties in standardized samples [8]. In vitro testing also offers such advantages as higher speed, greater accuracy, repeatability, and easy execution of experiments [17]. Extracted teeth also can be used as a die model to reproduce the actual force distribution in measuring fracture resistance [18]. However, it is not possible to standardize the preparation of teeth. The non-standardization of the preparation results in different cement thicknesses in each sample and this situation may increase stress within the restorative material and increase the likelihood of fracture [19]. In addition, physio-pathological changes may cause sclerotization in the dentin structure, which may result in decreased bonding protocols [20]. Therefore, the standardization of dentin–cement bonding strength may not be possible in studies using extracted teeth. In addition, it was stated that the epoxy die models did not show a significant difference from extracted teeth in determining fracture strength [21]. For this reason, resin die models, which show better fit and homogeneous cement thickness, were used in the present study.

There are not many prefabricated alternatives for aesthetic full crown restoration requirements in children. Commercial brand zirconia crowns may be overly expensive or even unavailable in some countries [22]. Therefore, the choice of these crowns may be limited. In these limited conditions, 3D-printed materials could present a new contribution to dentistry [23]. However, it has not yet been included in clinical practice in pediatric dentistry as much as prefabricated crowns. In this study, FRRCs, which are produced as an alternative to the disadvantages of zirconia crowns and 3D-printed crowns, were preferred.

Adhesive type of cement (e.g., glass ionomer cement [GIC], resin-modified glass ionomer cement [RMGIC], and resin cement) and non-adhesive luting agents (e.g., zinc oxide–eugenol cement, zinc phosphate cement, and polycarboxylate cement) are used for cementing crowns. Glass ionomer cement was preferred on cementation to provide maximum retention in the current study because of the recommendation of the FRRC manufacturer.

According to Centers for Disease Control and Prevention guidelines for sterilization, two common steam sterilization temperatures are 121 °C (250 °F) and 132 °C (270 °F). Recognized minimum exposure periods for sterilization of wrapped items are 30 min at 121 °C (250 °F) or 4 min at 132 °C (270 °F) [24]. The minimum exposure period for steam sterilization for unwrapped items is 20 min at 121 °C (250 °F) [25]. Although the manufacturer of FRRCs recommends autoclave sterilization at 132 °C, the sterilization method at 121 °C was preferred for unwrapped crowns in the current study, considering that high temperature autoclave sterilization may have negative effects on the color and microstructure of resin-based dental materials.

Color changes can be evaluated visually or using various instrumental techniques, such as a spectrophotometer and a colorimeter. Visual color assessment is unreliable because of inconsistencies in color perception. Instrumental measurements are widely used to measure color changes, as they eliminate subjective interpretation [26]. The reference threshold ΔE value is used to evaluate the results obtained according to color differences. Regarding the human eye’s ability to detect color differences, three threshold intervals have been proposed: ΔE < 1 not detectable by the human eye, 1 < E < 3.3 detectable by qualified operators and clinically acceptable, and ΔE > 3.3 detectable by patients and untrained observers and considered clinically unacceptable [16]. Consequently, the current study used the acceptability threshold of ΔE = 3.3. A recent study examined the color change of different brands of zirconia crowns after sterilization and found that although there was a slight perceived difference, there was no color change above the acceptability threshold in any group [27]. According to the results of this study, FRRCs showed color change above the acceptability threshold even after the first sterilization. And as the number of sterilizations increased, the color change increased even more. This color change may be related to the high pressure and heat generated during autoclave sterilization, causing degradation of the resin [28]. Accordingly, more care should be taken when determining the crown size of FRRCs. Avoiding unnecessary FRRC trials reduces the number of sterilizations that cause unacceptable color changes in crowns.

Masticatory forces in the mouth over a long period can cause fatigue, leading to crown fractures [8]. Fracture resistance testing cannot simulate the forces in the clinical oral environment. Nevertheless, this test can at least detect the fracture resistance differences between FRRCs that undergo different sterilization cycles. This testing method has been also widely used in previous studies [11, 19].

The physiological maximal occlusal force may vary according to facial morphology, occlusion, craniofacial dimensions, head posture, and age [29]. Owais et al. [30] evaluated the maximum bite forces in different age groups with different dentition periods and determined the maximum bite forces as follows: 176 N in the early primary dentition (mean age 3.37 ± 0.23 years), 240 N in late initial dentition (mean age 5.86 ± 1.15 years), 289 N in early mixed dentition (mean age 8.15 ± 0.67 years), 433 N in late mixed dentition (mean age 9.97 ± 0.86 years), and 527 N in permanent dentition (mean age 14.03 ± 2.14 years). In addition, a study evaluating the molar bite force according to Angle classification in children aged 7–13 found that the Angle Class II group showed the highest bite force (369.3 N) [31]. As for adults, several studies have reported that the mean physiological maximum occlusal force is 222–445 N (mean, 322.5 N) in the premolar region [32, 33]. A study investigating maximum bite force revealed that females with and without bruxism have no significant difference in bite force, whereas bruxist males have higher bite forces [34]. It has also been reported that the maximum bite force can exceed 110% in nocturnal bruxism compared with maximum daytime occlusal forces [35]. In this study, the mean fracture resistance of FRRCs following different sterilization cycles did not differ statistically (p = .055); however, overall, a tendency to decrease fracture resistance was observed as the sterilization cycle increased. This decrease may be due to the adverse effects of high-pressure steam during autoclave sterilization due to the breakdown of bonds in the resin matrix [13]. Moreover, repeated sterilization cycles may exacerbate this effect. The mean fracture loads of the FRRCs following different sterilization cycles were above the reported maximum chewing forces, although a decrease in fracture strength was observed. Therefore, it can be assumed that FRRCs withstand maximum chewing forces even if autoclaved.

Similar to the results of Yağcı et al. [13], who evaluated the effects of sterilization on fiber posts, autoclave sterilization increased the oxygen atom number on the crown surfaces in the present study. The drying cycle after autoclave sterilization may be responsible for the higher weight% of oxygen atoms on the surface of FRRCs.

In this study, autoclave sterilization, the commonly used sterilization method in clinics, was evaluated. However, EOG or chemical sterilization can also be used in crown sterilization. Not using different sterilization methods is considered a limitation of this study.

## Conclusions

According to the results of current study evaluating the effect of sterilization cycles on the chemical composition, color stability and fracture resistance of FRRCs;

- FRRCs demonstrated color variability above the acceptability threshold following the autoclave sterilization. The color change showed a rising trend with each sterilization cycle.
- Although not statistically significant, fracture resistance was decreased after each sterilization cycles.
- Avoiding unnecessary FRRC trials is important to prevent deterioration in the physical and chemical composition of crowns by reducing the number of sterilizations.

## Data Availability

The datasets and materials used or analysed during the current study are available from the corresponding author on reasonable request.

## References

[1] American Academy of Pediatric Dentistry (2016). Clinical Affairs Committee, and restorative Dentistry Subcommittee. Guideline on restorative dentistry. Pediatr Dent.

[2] Cohn C (2012). Pre-veneered Stainless Steel Crowns—An Aesthetic Alternative. Oral Health.

[3] Tote J, Gadhane A, Das G, Soni S, Jaiswal K, Vidhale G (2015). Posterior esthetic crowns in peadiatric dentistry. Int J Dent Med Res.

[4] Hartmann C (1983). The open-face stainless steel crown: an esthetic technique. ASDC J Dent Child.

[5] Krämer N, Rudolph H, Garcia-Godoy F, Frankenberger R (2012). Effect of thermo-mechanical loading on marginal quality and wear of primary molar crowns. Eur Archives Pediatr Dentistry.

[6] Prabhakar AR, Chakraborty A, Nadig B, Yavagal C (2017). Finite element stress analysis of restored primary teeth: a comparative evaluation between stainless steel crowns and preformed zirconia crowns. Int J Oral Health Sci.

[7] Donly KJ, Méndez MJC, Contreras CI, Liu JA (2020). Prospective randomized clinical trial of primary molar crowns: 36-month results. Am J Dent.

[8] El Makawi Y, Khattab N (2019). In vitro comparative analysis of fracture resistance of lithium disilicate endocrown and prefabricated zirconium crown in pulpotomized primary molars. Open access Macedonian journal of medical sciences.

[9] Yang B, Lange-Jansen H, Scharnberg M, Wolfart S, Ludwig K, Adelung R (2008). Influence of saliva contamination on zirconia ceramic bonding. Dent Mater.

[10] Freilich M, Meiers J, Duncan J, Goldberg A, Freilich MA, Meiers JC, Duncan JP, Goldberg AJ (2000). Rationale for the clinical use of fiber-reinforced composites. Fiber-Reinforced Composites in Clinical Dentistry Hanover Park.

[11] Garoushi S, Vallittu PK, Lassila LV (2007). Fracture resistance of short, randomly oriented, glass fiber-reinforced composite premolar crowns. Acta Biomater.

[12] The figaro crowns website. https://figarocrowns.com/pages/why-figaro-crowns. Accessed 27 June 2022.

[13] Yagci F, Ustun Y, Zortuk M, Agirnasligil M (2019). Effect of sterilization on bond strength and mechanical properties of fiber posts. J Adhes Dent.

[14] de Sousa-Lima RX, de Sousa Santos K, de Azevedo Silva LJ, de Freitas Chaves LV, Alonso RCB, Borges BCD (2022). Can sterilization methods influence surface properties of resin composites? A purpose for previewing bias in laboratory bacterial adhesion tests. Microsc Res Tech.

[15] Egilmez F, Ergun G, Cekic-Nagas I, Vallittu PK, Lassila LV (2018). Does artificial aging affect mechanical properties of CAD/CAM composite materials. J prosthodontic Res.

[16] Vichi A, Ferrari M, Davidson CL (2004). Color and opacity variations in three different resin-based composite products after water aging. Dent Mater.

[17] Salli KM, Ouwehand AC (2015). The use of in vitro model systems to study dental biofilms associated with caries: a short review. J oral Microbiol.

[18] Townsend JA, Knoell P, Yu Q, Zhang J-F, Wang Y, Zhu H (2014). In vitro fracture resistance of three commercially available zirconia crowns for primary molars. Pediatr Dent.

[19] Vinson L, McCrea M, Platt J, Sanders B, Jones J, Weddell A. Fracture resistance of full ceramic primary crowns. J Dent Oral Health Cosmesis 2016;1.

[20] Weber D (1974). Human dentine sclerosis: a microradiographic survey. Arch Oral Biol.

[21] Yucel MT, Yondem I, Aykent F, Eraslan O (2012). Influence of the supporting die structures on the fracture strength of all-ceramic materials. Clin Oral Invest.

[22] Hanafi L, Altinawi M, Comisi JC (2021). Evaluation and comparison two types of prefabricated zirconia crowns in mixed and primary dentition: a randomized clinical trial. Heliyon.

[23] Paradowska-Stolarz A, Wieckiewicz M, Kozakiewicz M, Jurczyszyn K, Mechanical, Properties (2023). Fractal Dimension, and texture analysis of selected 3D-Printed resins used in Dentistry that underwent the Compression Test. Polymers.

[24] Centers for Disease Control and Prevention. Available at: https://www.cdc.gov/infectioncontrol/guidelines/disinfection/sterilization/steam.html.Accessed 14 June 2023.

[25] Infection Prevention Guidelines. Available at: https://rwjms.rutgers.edu/research/core_facilities/ses/documents/SterilizationConcepts.pdf Acessed 14 June 2023.

[26] Chu SJ, Trushkowsky RD, Paravina RD (2010). Dental color matching instruments and systems. Review of clinical and research aspects. J Dent.

[27] Pate JD, Wells MH, Morrow BR, Ragain JC, Garcia-Godoy F (2021). Color stability of prefabricated pediatric zirconia crowns following sterilization. Pediatr Dent.

[28] Yilmaz Y, Guler C (2008). Evaluation of different sterilization and disinfection methods on commercially made preformed crowns. J Indian Soc Pedod Prev Dentistry.

[29] Al-Shibri S, Elguindy J (2017). Fracture resistance of endodontically treated teeth restored with lithium disilicate crowns retained. J Dent.

[30] Owais AI, Shaweesh M, Abu Alhaija ES (2013). Maximum occusal bite force for children in different dentition stages. Eur J Orthod.

[31] Sonnesen L, Bakke M (2005). Molar bite force in relation to occlusion, craniofacial dimensions, and head posture in pre-orthodontic children. Eur J Orthod.

[32] Komiyama O, Obara R, Iida T, Asano T, Masuda M, Uchida T (2015). Comparison of direct and indirect occlusal contact examinations with different clenching intensities. J Rehabil.

[33] Lin C-L, Chang Y-H, Pa C-A (2009). Estimation of the risk of failure for an endodontically treated maxillary premolar with MODP preparation and CAD/CAM ceramic restorations. J Endod.

[34] Karakis D, Dogan A (2015). The craniofacial morphology and maximum bite force in sleep bruxism patients with signs and symptoms of temporomandibular disorders. CRANIO®.

[35] Nishigawa K, Bando E, Nakano M (2001). Quantitative study of bite force during sleep associated bruxism. J Rehabil.

